# Core-Shell Processing of Natural Pigment: Upper Palaeolithic Red Ochre from Lovas, Hungary

**DOI:** 10.1371/journal.pone.0131762

**Published:** 2015-07-06

**Authors:** István E. Sajó, János Kovács, Kathryn E. Fitzsimmons, Viktor Jáger, György Lengyel, Bence Viola, Sahra Talamo, Jean-Jacques Hublin

**Affiliations:** 1 Environmental Analytical and Geoanalytical Research Group, Szentágothai Research Centre, University of Pécs, Pécs, Hungary; 2 Department of Geology and Meteorology, University of Pécs, Pécs, Hungary; 3 Department of Human Evolution, Max Planck Institute for Evolutionary Anthropology, Leipzig, Germany; 4 Department of Prehistory and Archaeology, Institute of History, University of Miskolc, Miskolc-Egyetemváros, Hungary; 5 Department of Evolutionary Genetics, Max Planck Institute for Evolutionary Anthropology, Leipzig, Germany; University of Kansas, UNITED STATES

## Abstract

Ochre is the common archaeological term for prehistoric pigments. It is applied to a range of uses, from ritual burials to cave art to medications. While a substantial number of Palaeolithic paint mining pits have been identified across Europe, the link between ochre use and provenance, and their antiquity, has never yet been identified. Here we characterise the mineralogical signature of core-shell processed ochre from the Palaeolithic paint mining pits near Lovas in Hungary, using a novel integration of petrographic and mineralogical techniques. We present the first evidence for core-shell processed, natural pigment that was prepared by prehistoric people from hematitic red ochre. This involved combining the darker red outer shell with the less intensely coloured core to efficiently produce an economical, yet still strongly coloured, paint. We demonstrate the antiquity of the site as having operated between 14–13 kcal BP, during the Epigravettian period. This is based on new radiocarbon dating of bone artefacts associated with the quarry site. The dating results indicate the site to be the oldest known evidence for core-shell pigment processing. We show that the ochre mined at Lovas was exported from the site based on its characteristic signature at other archaeological sites in the region. Our discovery not only provides a methodological framework for future characterisation of ochre pigments, but also provides the earliest known evidence for “value-adding” of products for trade.

## Introduction

Ochre is the colloquial term used by archaeologists to describe an earth or rock containing red or yellow oxides, most commonly hydroxides of iron [1, 2]. Red ochres typically consist of iron oxides (Fe_2_O_3_) derived from hematites (from the Greek word for “blood-like”) and other iron-rich rocks. Red ochres are relatively common in natural geological and soil formations.

Ochres have long been used for a range of applications by prehistoric people. The best known examples of Palaeolithic use of ochre are from cave paintings [3] and ritual burials [1]. From the Upper Palaeolithic record, red ochres are best known for their use in cave paintings and ritual burial contexts. Although early use of red pigment has long been associated with early modern humans in Africa and the Near East [2, 4–6], ochre has also been associated with European Neanderthal sites, the earliest securely dated evidence for which dates to 200–250 ka for the early Middle Palaeolithic Neanderthal site in Maastricht-Belvédère [7].

Potential symbolic purposes of ochres are demonstrated by present-day hunter-gatherers, yet remain hypothetical or circumstantial in the case of Palaeolithic use. Ochres are used by modern hunter-gatherers for both internal and external medications, food and wood preservatives, insect repellents, and for tanning of hides [1, 3, 8–12]. Circumstantial evidence of ochre use in Palaeolithic sites is limited to burial customs; the closest known use of ochre in Palaeolithic burials relative to our study area occurs in the Pavlovian archaeological context in Lower Austria and Moravia, and dates to 30–33 k cal BP [13]. The ancient use of iron oxides for “symbolic” purposes should be viewed as a hypothesis that remains to be tested, rather than simply assumed [11].

Regardless of whether ochres have long held a symbolic purpose, early evidence of intentional processing of raw ochres into higher value products has been demonstrated by identification of ochre powder as an ingredient in the ancient manufacture of compound adhesives [14]. In Europe, the use of ochre as processed mineral pigments started in the Palaeolithic [7, 15, 16]. Rock paints are the most spectacular in the epoch of the Magdalenian (ca. 17–12 ka), with famous paintings in the rock caves of Lascaux (Dordogne, France), Niaux (Ariége, France) and Altamira (Santander, Spain) [17].

The study of prehistoric ochre has mainly been focused on the analysis of raw materials and their uses [1, 18–23]. Numerous methods have been successfully tested to determine the nature and the provenance of the raw materials, such as X-ray diffraction (XRD), X-Ray fluorescence (XRF), FTIR and Raman spectrometry, scanning electron microscopy coupled with energy dispersive X-ray spectrometry (SEM-EDX), proton induced X-ray emission (PIXE), inductively coupled plasma mass spectrometry (ICP-MS), or instrumental neutron activation analysis (INAA) [24–28].

The natural sources of prehistoric ochres are not easily identifiable due to the ubiquity of raw material sources, combined with high mineralogical variability. In this study we address this challenge to provenancing raw ochre sources by investigating the crystallographic and microstructural features of archaeological pigments, in order to identify the actual origin of red pigments based on hematite. Previous hypotheses regarding the provenance of archaeological ochres have typically been based on geographical proximity rather than considering their mineralogical and microscopic-scale features, which require a mineralogical or materials science approach. The fundamental characteristics of natural and artificial hematite must, however, be taken into account in order to fingerprint an ochre source. For example, hematite can be obtained artificially by heating the mineral goethite to moderate temperatures (300–350°C) [19, 23, 29], which lies well within the range of Palaeolithic campfires. Considering this technological implication, it is not surprising that a number of research efforts have focussed on identifying a reliable method to discern natural from artificially-produced hematite from goethite heating [29, 30].

Here we put forward an alternative hypothesis for pigment production: the “core-shell” technique. We suggest that pigments were not only derived from pure natural ochre or heat-treated goethite, but also that prehistoric people ground the highly pigmented red material to produce finer particles, and then exploited natural oxidation processes whereby the fine hematite nano-particles—the “shell”–coated non-pigmented sand or silt-sized grains—the “core”. This process would have resulted in substantially higher volumes of pigmented material than was available by using the pure ochre. Most modern-day core-shell nanostructures are developed with the idea to combine two materials—and thus two properties—within one structure. In this way, the nanostructure influences the properties of both core and shell, offering a variety of new possibilities and combinations, and expanding the availability of ochre for a wider range of activities. A typical example of modern core-shell pigments are those which contain a core of cheap oxides covered with a layer of phosphates, which are produced as a more economically feasible solution for anticorrosive pigments than pure pigment [31]. The core-shell processing technique would therefore have been an effective and efficient way to enhance the output and value of ochre products in trade.

The core-shell hypothesis is best investigated by investigating at the microscopic scale the mineralogical and internal structure characteristics of ochre from a known raw material source, in order to establish its formation, provenance, intentional post-extraction processing. Of further interest is its antiquity, to determine precisely how early the method was adopted as a means of “value-adding” a raw material product and therefore better understand how long people have been employing enterprising and innovative means of improving products for trade.

We use the Hungarian ochre quarry site of Lovas as a case study for our approach. This is the second known instance of Palaeolithic ochre exploitation in eastern central Europe, following Rydno in Central Poland [32]. Because traces of Palaeolithic ochre mining are so sparse in this region, while nevertheless ochre was widely used, the characterization of the Lovas pigment may help identify the origin of ochre in archaeological sites in central Europe. In Hungary, the earliest known use of red pigment was found on a polished mammoth tooth plate recovered at the Middle Palaeolithic site of Tata [33], and dated to the Last Interglacial (125–90 ka) [34]. Besides this exceptional instance, ochre is most frequently found at sites associated with the Ságvárian and Epigravettian cultures of the Late Upper Palaeolithic (ca. 20–10 kcal BP). As yet, nothing is known of the characteristics of the red pigment derived from the Lovas quarry, however clearly it is of interest to determine when mining activities took place, who undertook them, and to characterise the pigment produced by the Lovas mine.

In this study we clarify the age of mining activity at Lovas with a new set of radiocarbon dates, characterize the petrographic and mineralogical attributes of the Lovas core—shell type red ochre, and establish a database for further investigations of the distribution of processed material throughout the region coeval with its extraction. X-ray diffraction (XRD) is used to identify and characterise the pigments [35, 36].

## Results

### The Upper Palaeolithic ochre quarry at Lovas

**Fig 1:**
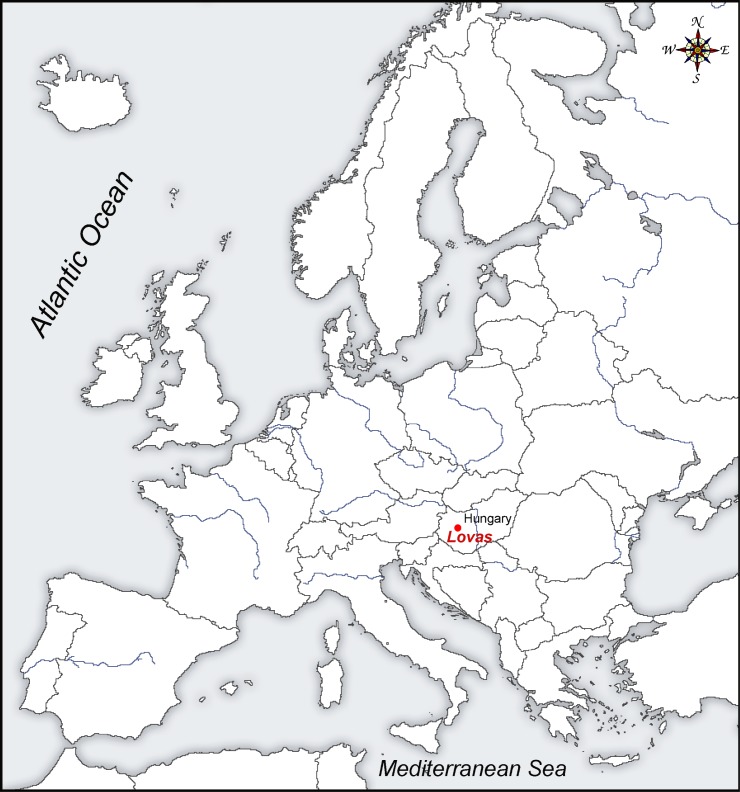
Location of the Lovas red ochre mine in Hungary.

**Fig 2:**
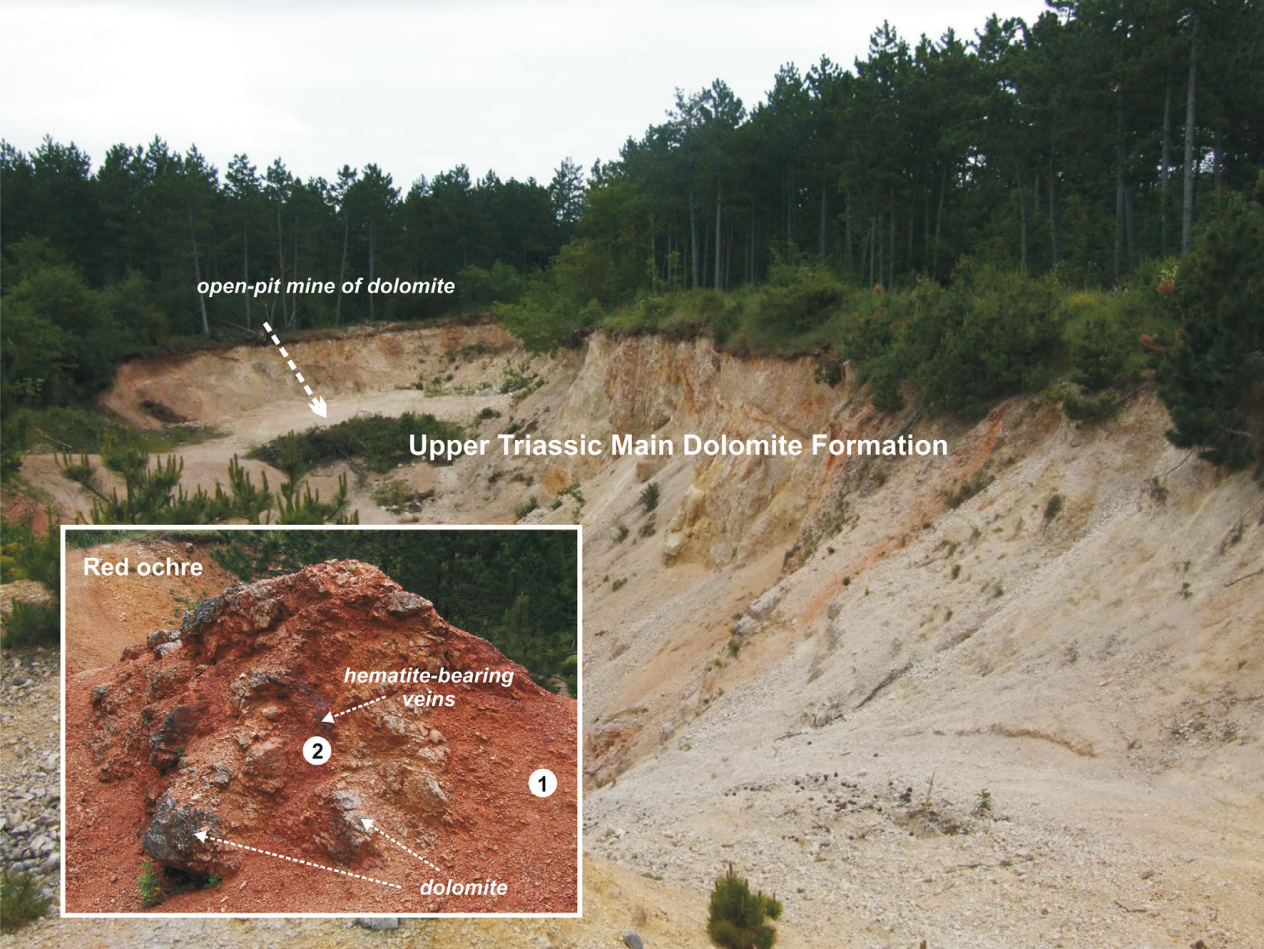
Geological context of the site located within Upper Triassic dolomite bedrock. Close-up view of the red ochre in the dolomite breccia (bracketed), white dots are the sampling points.

The Lovas quarry is located 3 km north of Lake Balaton in central western Hungary, in a shallow basin immediately behind the bedrock-dominated Balaton Highland. The small quarry was operated for dolomite grit until the 1960s (Figs 1 and 2), and lies 200 m off the Veszprém-Csopak road, in the vicinity of Lovas village. The Main Dolomite Formation is the thickest and most widespread Triassic formation in the region and accumulated beneath the Tethys Sea [37]. Today it underlies large areas of the Balaton Highland and its hinterland, where the quarry is located [38]. The Main Dolomite comprises nearly pure dolomite, with only the supratidal facies member containing thin clay and marl interbedding [39, 40]. The site lies adjacent to extensive deposits of loess derivates and sandy loess associated with the Danube and Drava catchments [41], and which preserve valuable evidence for past climatic conditions prevailing during the period of exploitation of the ochre mine [42, 43].

Ochre has formed within the dolomite as rare thin, yellow, yellowish red or red marly dolomite with thin calcite veins (Fig 2). The ochre is a fine-grained (silt and clay) sediment and its colour is red (Munsell soil colour 2.5YR 4/6 or 10R 4/6).

The outcropping of iron ore—ochre—veins within the dolomite at the Lovas locality drew the attention of Palaeolithic people who exploited these veins for ochre production. The main prehistoric mine site follows one of these veins, forming a pit approximately 6 meters long, 2.5 m deep, and 2.5 m wide. Approximately 25 m^3^ of ochre was mined [15]. Rubble within the pit comprises overburden from Palaeolithic mining activities, and comprises a dark-red, crumbly sediment (herewith termed “ochre grit”), most likely derived from the weathered local bedrock, and mixed with the ochre material [15].

Archaeological traces found within the rubble infill from the prehistoric ochre mining pit provides evidence both for ochre mining and processing methods, as well as for the antiquity of mining activities. Archaeological traces include antler picks and shovels, stones for grinding the ore, and tubes made from hollow bones which are interpreted to have acted as carrying tubes to transport the powder produced from mining activities [15]. Of the hollowed tubes, we were unable to extract ochre material for sampling. However, where bone tools were found with ochre traces on their surface have been preserved; we were able to sample very fine (<10 μm) ochre for our study.

**Fig 3:**
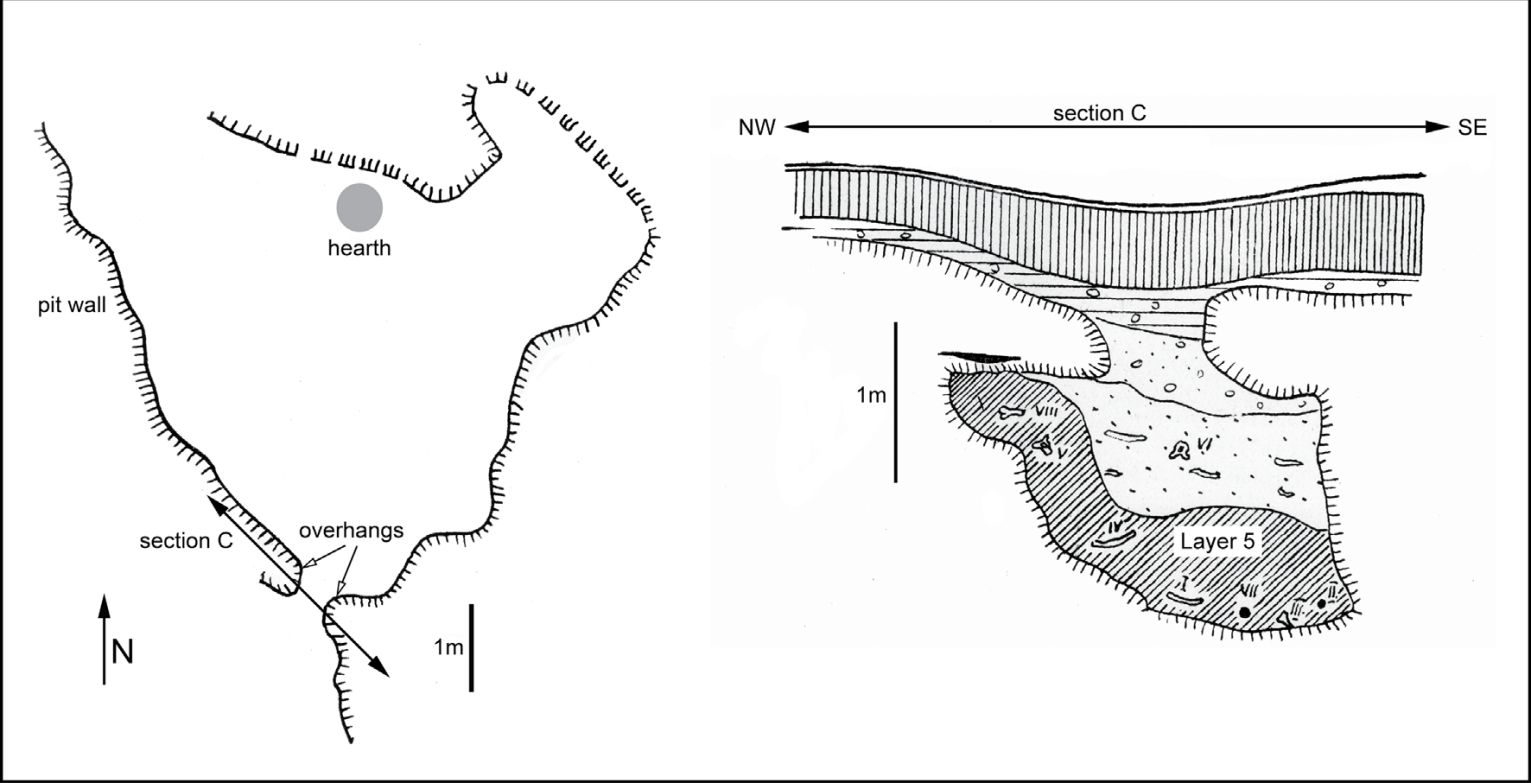
Plan (left) and stratigraphy of Pit 2 (right) (after [15]).

**Fig 4:**
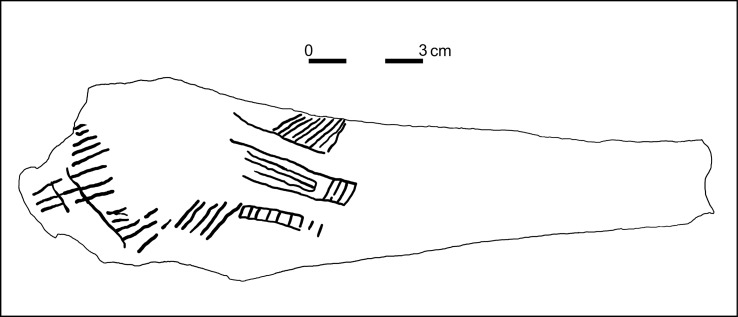
Decorated elk ulna from Pit2 Layer 5 (after [15]).

A large proportion of the archaeological assemblage comprises bone remnants and bone tools. A number of worked elk bones [44, 45] and eight fragments of antler [46] were identified to species level from the assemblage recovered from the ochre mine [47]. 97 complete and broken bone tools [46], and 21 stone artefacts [45] were excavated from pit 2, layer 5 (70–80 cm thick) [15] (Fig 3). This layer immediately overlies the bedrock. Of the 139 bones, 70% are tools [45]. The prime bone tool raw material is *Alces* (Eurasian elk), comprising 80% of the tools when excluding six items indistinguishable from *Cervus elaphus* (red deer). Elk skeletal parts in the toolkit are derived from ribs, ulna, metapodial, metacarpal, antler, tibia, humerus and femur [45]. The ulna items in the tool kit were heavy duty mining tools, compared with the metacarpal bones which were more likely used for finer ochre extraction. Red deer bone tools comprise eight ribs, two antlers, and one tibia. Besides mining, the bone tools served also to perforate and scrape, according to macroscopic usewear traces [45]. In addition, there is a single reindeer antler evidently used as a precursor, and a pointed ibex metapodial. One bone tool made from elk ulna has a decoration of engraved lines (Fig 4), although this ornament cannot be specifically associated with a particular archaeological culture. One of the bone tools is a bipoint [15], the shape of which is typical of the Late Upper Palaeolithic of Central Europe. In the unworked bone collection (N = 42), elk makes up 45%, red deer 36%, and there is 12% indistinguishable fragments between elk and red deer. Single bone fragments from a small horse and a crane were also identified [44, 45]. Boar remains were also found outside pit 2 [15]. The taphonomic study of the complete faunal assemblage indicates a mixture of worked and unworked bones (N = 139) which were not derived in situ, but rather were brought to the site with the intention of using them for a variety of purposes associated with ochre extraction and processing [45].

The lithic assemblage is typologically inconsistent, which led to the conclusion that the mine was operated by different lithic “cultures” over a long period of time. The lithic assemblage is dominated by blank flakes of relatively fresh radiolarite. One patinated and relatively weathered bifacial tool, attributed to the Late Middle Palaeolithic Jankovichian period [47], is formed from hydro-quartzite [45]. The variability in raw material and in stone tool surface condition may refer to a difference in age of the lithic finds, which led to the early conclusion that the mine was worked over a long period of time. The assessment of the chronological position and the cultural affiliation of the Lovas quarry site have changed several times since its discovery, with interpretations alternating between the Last Interglacial and the end of the Pleistocene [15]. This was due to the lack of radiocarbon dates and the variability of the lithic assemblage. The timing of ochre processing at the site was addressed in this study by new radiocarbon dates from the bone tools, as described in the section below.

### Antiquity of ochre processing at the Lovas Quarry

Until now the antiquity of mining activity at Lovas was interpreted based on the faunal assemblage, a single ^14^C date, and stone tool typology based on a single bifacial leaf-shaped tool characteristic of the Late Middle Palaeolithic Jankovichian culture [47]. These interpretations suggested that mining activities extended from last Interglacial Neanderthal occupation and continued by Anatomically Modern Humans until the onset of the Neolithic [15, 44–47].

In this study, radiocarbon dating was undertaken on 5 bone artefacts collected from the rubble overburden pit 2, layer 5, in order to establish the antiquity, and if possible the duration, of ochre mining activities at Lovas. The bone fragments taken as samples were all from *Alces* sp. (Eurasian Elk)–the most common raw material—were derived from the same stratigraphic unit as all other bone fragments, and bore clear impacts of human activity. Since the different samples were weathered to varying degrees, it was assumed that the resulting chronology would reflect the full duration of mining activities at Lovas, and that this may have been of significant duration. No dry bone breakage was reported by the taphonomy study [45] and therefore we do not expect significant post-depositional disturbance to the assemblage and archaeological record at pit 2.

**Fig 5:**
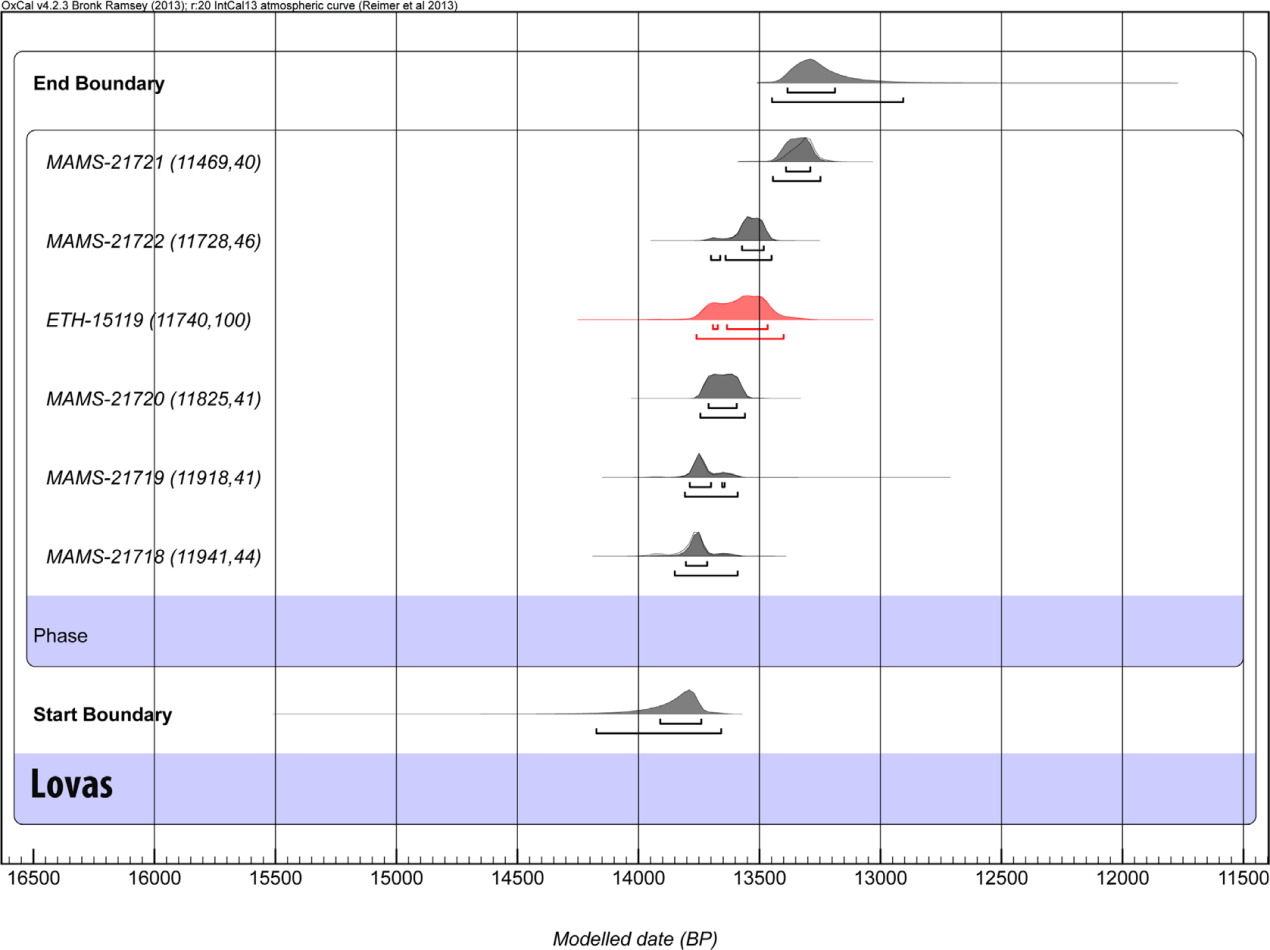
Calibrated ages and boundaries. Calibrated ages and boundaries calculated using OxCal 4.2.3 [86] and IntCal13 [87]. Lovas ages are in black and the previous radiometric result from ETH is in red.

The uncalibrated radiocarbon dates range from 11,941±44 to 11,469±40 ^14^C years BP (1σ error). These results are in full agreement with the previous radiocarbon date on elk bone (*Alces alces* L. 1758) produced by the Eidgenössische Technische Hochschule (ETH) Zurich, which yielded a date of 11,740±100 ^14^C BP [45] (Fig 5).

The calibrated radiocarbon dates yield a modelled range for the duration of mining activities of between 13.2–13.8 kcal BP (Fig 5 and Table 1). These results suggest that mining activities, occurring over approximately 600 years, were of relatively short duration. This contradicts previous hypotheses regarding a long period of ochre mining at Lovas, based on the single anomalous presence of an apparently Middle Palaeolithic stone tool made of a different raw material to the other lithics. It is most likely that this tool represents a singular anomaly and was brought to the site at a date substantially later than its manufacture, and cannot be used to argue for Neanderthal ochre exploitation. It could have been collected and used by the late Palaeolithic population that started extracting ochre at Lovas around 13.8–13.2 kcal BP. The period of ochre mining at Lovas corresponds to the Allerød interstadial GI-1b-d [48].

**Table 1:** Modelled and Unmodelled calibrated results at Lovas, provided by OxCal 4.2.3 [85] using the International Calibration Curve IntCal 13 [86].

	Unmodelled (BP)				Modelled (BP)			
Indices Amodel 98.5 Aoverall 97.2	from	to	from	to	from	to	from	to
	*68*.*2%*		*95*.*4%*		*68*.*2%*		*95*.*4%*	
**End Boundary**					13,390	13,180	13,450	12,900
MAMS-21721 (11469,40)	13,370	13,270	13,420	13,230	13,390	13,290	13,450	13,240
MAMS-21722 (11728,46)	13,580	13,480	13,710	13,450	13,580	13,480	13,700	13,450
ETH-15119 (11740,100)	13,700	13,460	13,770	13,380	13,700	13,460	13,760	13,400
MAMS-21720 (11825,41)	13,710	13,590	13,750	13,560	13,710	13,590	13,750	13,560
MAMS-21719 (11918,41)	13,790	13,700	13,830	13,580	13,790	13,640	13,810	13,590
MAMS-21718 (11941,44)	13,820	13,720	13,960	13,610	13,810	13,710	13,850	13,590
**Start Boundary**					13,910	13,740	14,180	13,650
**Lovas**								

### Characterisation and provenance of the Lovas ochre

The Lovas ochre was characterised based on sampling from the Palaeolithic ochre grit found on the quarry floor, the in situ red dolomite veins, and from ochre traces next to a bone tool found at the site. The three types of samples were investigated in detail by means of X-ray diffraction (XRD) and microscopic analyses.

**Fig 6:**
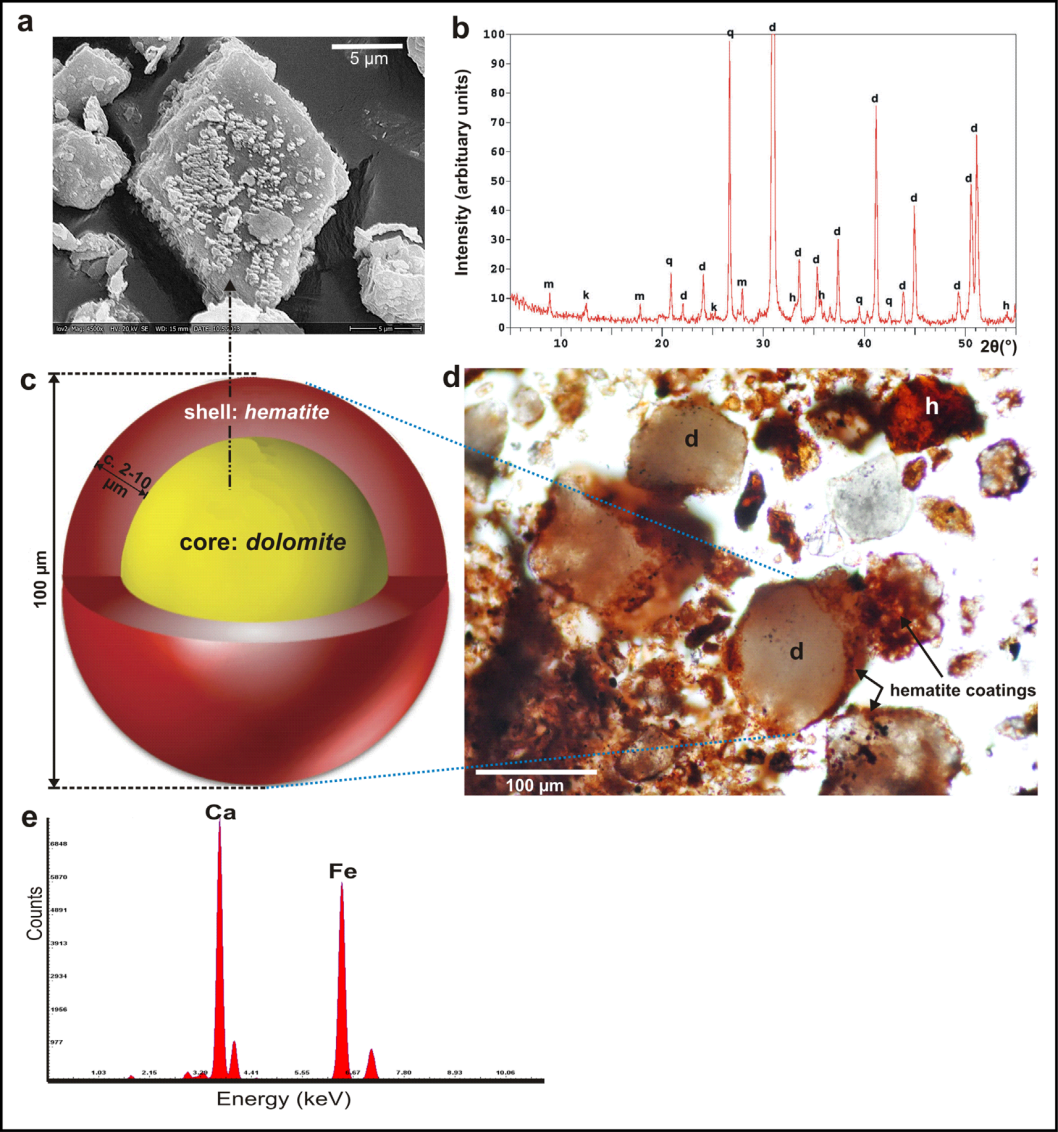
Characteristics of the Lovas red ochre. (a) SEM images of hematite-coated, rhombohedral dolomite crystal. (b) XRD analysis of the red ochre sample; m—muscovite, k—kaolinite, d—dolomite, h—hematite, q—quartz. (c) Model of core-shell particle (after [88]). (d) Photomicrograph of red ochre sample; d—dolomite, h—hematite. (e) Spectra showing identification of Ca and Fe by SEM—EDS.

Our quantitative evaluation of XRD spectra shows that the ochre mineralogy is dominated by dolomite (about 80 w/w %), with smaller proportions of quartz (~10%), clay (~5% kaolinite) and mica (~1% muscovite). The mineralogical characterisation of the ochre by XRD is summarised in Fig 6b, and shown in more detail in S1 Fig and S1 Table. The XRD spectra show that the mineralogy of the in situ red dolomite veins is dominated by dolomite (see S2 Fig). By contrast, the XRD spectra of very fine (<10μm) particles of red ochre from next to the bone tool (S3 Fig) show that the fine ochre is dominated by dolomite (c. 70 w/w %), with smaller proportions of quartz (~6%), clay (~8% kaolinite) and calcite (~3%). Hematite appears in greater proportions (~12%) in individual crystal particles from the bone tool ochre (see S4 Fig and S2 Table).

The natural pigments do not dominate, but instead are intermixed with clays and quartz [49]. The intensive red colour of the ochre is derived from relatively small proportions of hematite (~5%). The small proportion of hematite relative to the intensity of the ochre’s colour was unexpected.

**Fig 7:**
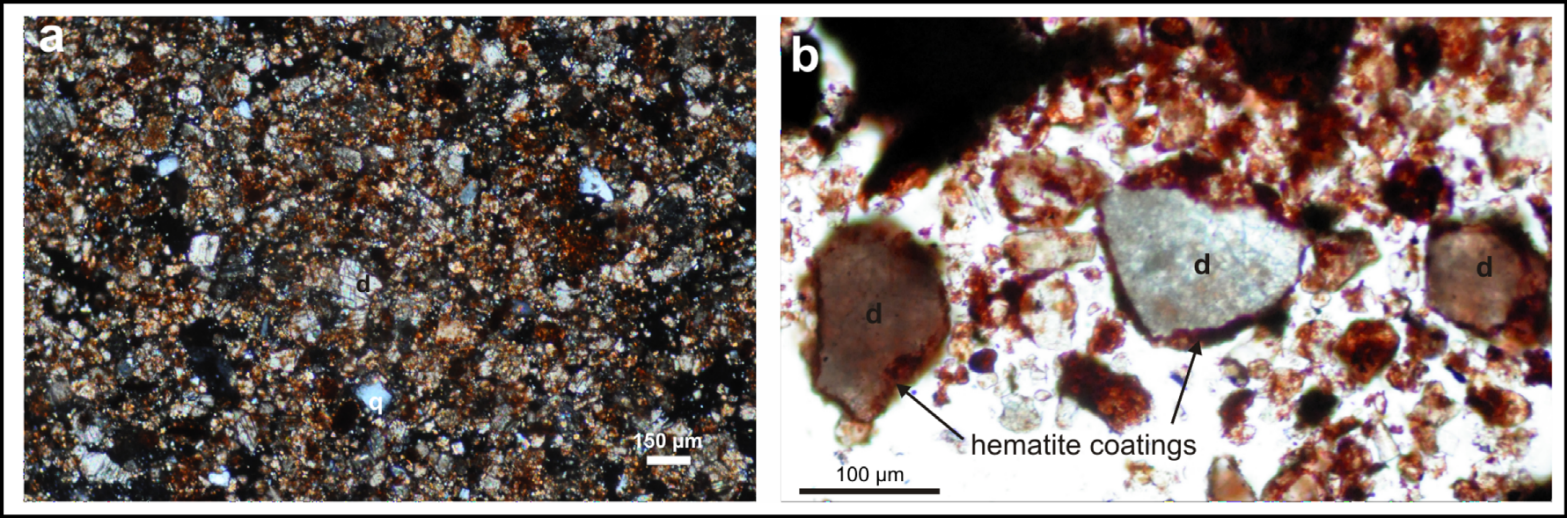
Photomicrographs of the Lovas red ochre samples. (a) Angular morphology of the coarse grains in red ochre (cross-polarized light). (b) Hematite-coated mineral (mostly dolomite) grains (plane polarized light); d—dolomite; q—quartz.

In order to determine how such a small proportion of hematite can dominate the ochre material, the internal structure of the pigment was investigated at microscopic level. Polarization microscopy shows that the majority of ochre mineral grains are finer than 150 μm in diameter, mostly ranging from 50 to 150 μm (Fig 7), and comprise dolomite and quartz. Grains <50 μm represent a very small fraction, comprising clay, amphibole and hematite particles. Most of the coarser-grained quartz and dolomite grains which dominate have irregular and angular shapes (Fig 7). Scanning electron microscopy (SEM; equipped with an energy-dispersive spectrometer) analyses of the ochre reveal the internal structure of ochre fragments. The ochre comprises Ca (52.1 wt%) and Mg (4.2 wt%) as dolomite, partly coated by Fe (22.7 wt%) as hematite (Fig 6e; S3 Table). A red staining agent surrounds the quartz and dolomite grains of the sedimentary matrix. This occurs as a very thin coating, and occasionally along the cleavage face of the dolomite grains (Fig 6d). The thickness of the grain coatings ranges from <1 mm to tens of micrometres. Individual reddish crystal aggregates up to 50 μm (e.g., hematite) are also visible, but rare (Fig 6d).

At the sub-micron scale, SEM images of the dolomite surfaces show hematite as bright particles and aggregates sitting on the darker gray dolomite crystal surface (Fig 6a). Hematite particles are sphere/rhomboid like, with 0.1–2.0 μm diameter. The SEM images also confirmed that iron oxide coatings are not of uniform thickness, nor do they form as evenly distributed films, but rather as discrete sub-micron particles that spread across the dolomite and quartz surface (S4 and S5 Figs).

The ochre therefore can be characterised as having a *core* of dolomite, and occasionally quartz grains, with an external *shell* of pigment. This core-shell structure of the ochre grains is unlikely to have been produced naturally, since it contrasts with the more consolidated nature of the in situ pigment. The coarser core grains are angular, suggesting post-depositional fragmentation, most likely due to post-extraction processing of the raw ochre material. The distribution of the pigmented shell hematite, which gives the grains there colour, also suggests post-depositional redistribution of the naturally occurring fine hematite. This raises the question as to how this process was undertaken, and why.

## Discussion and Conclusions

### Environmental conditions during Lovas mining activities and implications for human behaviour

Mining activities at Lovas appear to have taken place over a relatively short period of time, from ca. 13.8–13.2 kcal BP. This period corresponds to the Allerød interstadial 1b-d. Comparison of the timing of this site with palaeoenvironmental and archaeological records enables hypotheses to be formed with respect to the implications for human behaviour of the mining activities at this particular site.

The archaeological record at the time of the Allerød interstadial 1b-d is as yet poorly understood in the Carpathian basin. An Epigravettian site at Nadap, northeast of Lovas between Lake Balaton and the Danube, yielded a date of 15.6 kcal BP from a horse phalange [50]. At Szekszárd-Palánk, south of Lovas and close to the Danube, radiocarbon dating on charcoal constrained the antiquity of an archaeological site to 12.0 kcal BP [51], although the cultural context of this site is as yet unclear. In the northern Carpathian basin from Hungary to Slovakia, there are no archaeological remains dated later than 21.0 kcal BP [52]. Evidence for Palaeolithic activity beyond the mining at Lovas must be sought from further afield, in the Czech Republic and Poland [53, 54]. Epi-Magdalenian occupations, postdating the latest Magdalenian, in Moravia at Kůlna Cave (layers 3 and 4) indicate occupation of that site around the same time as mining activities at Lovas (14.0 kcal BP) [55]. The dating at Kůlna was undertaken on *Alces* bones, as was the case for Lovas, although there is otherwise no cultural similarity between the sites. In Poland, Magdalenian radiocarbon dates are mostly no later than 14.0 kcal BP except for a few instances which are contemporaneous with Lovas [56, 57]. From the period after the Magdalenian in South Poland, Arched-backed Point Technocomplex (ABPT) lithic assemblages were recovered, though poorly dated by radiocarbon [58]. Despite the similar ages of these sites, the uncharacteristic lithic tools of Lovas cannot be related with the contemporaneous ABPT and the Epi-Magdalenian of those other sites.

The environmental conditions prevailing in the region during the Allerød interstadial 1b-d are best known from the surrounding loess records [41, 42], and from geomorphic information. This period represented a phase of relatively milder conditions postdating the last glacial maximum (LGM) and immediately preceding the short, cold Younger Dryas event [48]. LGM conditions in the Pannonian basin of the middle Danube catchment were cold and dry [42, 59, 60], dominated by steppe environments [60–62], and windy as evidenced by a peak in loess accumulation at the time [63–66]. However, the LGM was comparatively milder in the Pannonian basin than in the surrounding regions, and in western Europe, with the suggestion that this region acted as a refugium for thermophilic biota [67, 68]. The ensuing interstadial phase which oversaw mining activities at Lovas was slightly milder, yet remained cooler than the present day [60]. Loess accumulation decreased, suggesting less windy conditions [63]. Lake Balaton, adjacent to the Lovas site, was a chain of freshwater ponds which would later fill from west to east [69], but nevertheless providing a consistent water supply locally. The relative change in climatic conditions from the LGM to Interstadial 1b-d in this region is, however, less extreme than in the adjoining regions outside of the Pannonian basin. Consequently, it is feasible to suggest that if the Pannonian basin represented a refugium during the LGM, the subsequent interstadial opened up the adjoining areas, facilitating a widening of territories for hunter-gatherers by the time Lovas began to be exploited for its ochre resources.

The absence of archaeological sites contemporaneous with Lovas in the Carpathian basin, combined with the change in climatic conditions described above, may indicate that the area was abandoned during the Allerød climatic amelioration in favour of a wider territory beyond the Pannonian basin. Based on this hypothesis, the hunter-gatherer population would have visited the area solely in order to exploit mineral dyes without including the territory in their usual foraging range. Mining activity at Lovas was of short duration, but was nevertheless intensive.

### Implications of the mineralogical investigations for provenancing archaeological ochres

This study uses petrographic techniques to provide the first evidence for post-depositional treatment of ochres during the late Palaeolithic. Previous investigations interpreted the red material from Lovas as being not hematite, but limonite [15], a yellow-brown iron oxyhydroxide, which had been fire-heated to produce red ochre [70, 71]. The iron oxide content of unrefined, natural pigments is proportional to the intensity of the red colour; yellow ochres range from 10–50% FeO_total_, whereas red ochres may exceed 90% FeO_total_ [21, 49]. Our investigation yielded ~5% hematite (S1 and S3 Tables) and ~12% hematite (S2 Table), and therefore contradicts earlier interpretation that the ochres consisted of limonite. We demonstrate that the red material is in fact hematite, with a characteristic core-shell structure from which it can be deduced that a different post-extraction processing technique was applied.

There is in fact no evidence to suggest that heating played a role in the processing of the raw ochre. The red coloration is, in our case, due to hematite coating of quartz and dolomite grains. The two most common low-temperature iron(III) oxides on Earth are goethite (α-FeOOH) and hematite (α-Fe_2_O_3_). In general, the goethite and hematite responsible for red coloration of rock particles form by combined hydrolysis and oxidation of Fe(II) bearing minerals (e.g. pyrite, marcasite or iron-rich silicates), which afterwards dehydroxylate into hematite [49]. The iron cations within the microcrystalline hematite crystals used in the development of the ultrafine pigment found at Lovas were principally derived from the breakdown of iron-bearing detrital grains. Most likely the hematite formation at Lovas resulted from hydro-geochemical processes associated with low temperature groundwater through-flow, such as silicate dissolution, sulphate reduction and pyrite formation, and pyrite oxidation and formation of hydrous ferric oxides [49].

Since the core-shell structure of the Lovas ochre does not occur naturally, we need to seek a process-based explanation for how this structure was obtained. Iron oxide coatings can strongly bond to quartz surfaces through robust chemical bonding [72]. Therefore, iron oxide coatings do not form evenly distributed films of uniform-thickness, but rather form as discrete, submicron-sized particles distributed across sand surfaces. Individual hematite agglomerates may point to local precipitation of the iron oxides, rather than transport of colloidal iron oxides to the site. It is well known that dissolved Fe(III) is readily absorbed by quartz surfaces in near-neutral pH water [73], and that when an Fe(II)-bearing fluid enters an oxidizing zone, precipitation is expected. This is because of the reduction in solubility that accompanies oxidation [74–76]. Transport of dissolved Fe(III) is severely limited by low solubility and by the sequestration of dissolved Fe(III) by sorption to quartz grain surfaces. Thus, growth of iron oxide minerals is likely to be local and to involve the transport of iron as dissolved Fe(II).

### Evidence for “value-adding” of ochre as a product for trade: the core-shell method

The core-shell structure of the Lovas ochre is known from materials science. Core-shell theory presents a way to process low-grade raw materials, resulting in high performance, efficiently produced pigments with concomitant savings [77, 78]. The theory is based on depositing a surface layer of more valuable, anticorrosive pigment on a less valuable, more common extender material. The combination of both core and shell compounds (Fig 6c) produces new pigments with improved properties different from each of its individual components. This results in a higher yield of pigmented material using efficient processing methods. In the case of the Lovas ochre, the core material is represented by the coarser dolomite and quartz grains, with the shell provided by the finer grained, intensely coloured hematite.

The Lovas core-shell pigment represents the earliest-known incidence of this kind of efficient post-extraction processing, which would have produced a greater yield of pigment than otherwise available from simply extracting the raw material. This new method of pigment preparation combines the properties of components both readily available at the Lovas mine, in an economically feasible way. The bulk pigment produced at Lovas contains only 5–12% Fe_2_O_3_, compared with the 90% Fe_2_O_3_ typical of pure hematite ore pigment. Consequently it represents a form of “value-adding” to an already valuable, widely used product, providing higher gain in trade.

## Materials and Methods

No specific permissions were required for sampling at this locality, since it is on public land. Field studies were solely focused on collecting geological samples and therefore did not involve endangered or protected species.

At Lovas, the ochre outcrops are associated with clay and marl-interbedded dolomite layers that are intensely fractured locally due to the presence of faults. In the fractured areas, the dolomite unit exhibits intense red and brown colours (Fig 2). Unfortunately, by the time of sampling, the original pits had been eroded and removed from the sedimentary outcrop, although the ochre grit which accumulated on the quarry floor during Palaeolithic ochre mining is well preserved (Fig 2 point 1).

In this study, ten samples of ochre were collected from the Lovas site; five from fresh red ochre gritbelieved to be the result of prehistoric human activity (Fig 2. point 1), and five from undisturbed in situ dolomite veins likely to have been related to the former pit walls (Fig 2. point 2). In addition, one sample was of fine ochre particles near a bone tool. Geologically, all the samples are considered to be derived from the Upper Triassic Main Dolomite Formation.

In the laboratory, the ochre samples were first dried at 100°C in an oven overnight, to remove moisture. The dried samples were separated for thin sections and ground into powder using an agate mortar and pestle.

### X-Ray diffraction analysis (XRD)

Powder X-ray diffraction was used to identify the different crystalline phases present in pigments. Spectra were collected on a Philips PW 3710/PW1050 Bragg-Brentano diffractometer using CuKα radiation (λ = 1.54186 Å), with a proportional detector containing a graphite monochromator in the reflected beam. The following analysis parameters were used: 40 kV, beam current of 35 mA, scan range of 4°–70° 2θ in continuous scan of 0.02° 2θ steps, with 1 s per step. The diffraction spectra were compared with the International Centre for Diffraction Data database and evaluated for quantitative phase composition using full pattern fit software.

### Scanning electron microscopy (SEM) and element analysis

Individual ochre particles were investigated using a field-emission scanning electron microscope (JEOL JSM-5800), fitted with an energy-dispersive X-ray analysis (EDS) to distinguish different mineral particles and their shape, size and elemental composition.

### Petrographic study

Vacuum impregnation was used for the soft ochre samples. The dried samples were put into mounting cup holders and then placed in a vacuum impregnation unit (Struers CitoVac). The mounting cups in the vacuum chamber were filled with impregnation material (EpoFix resin) under vacuum. Specimens impregnated with resin were then bonded onto a glass slide. Transmitted light microscopy and imaging was performed on polished thin sections using a Nikon Eclipse E600 POL microscope fitted with Nikon D90 digital camera.

### Radiocarbon dating

The five bone samples (Pb.53/96.3 (100); Pb.53/98.2; Pb.53/97.3 (157); Pb.53/99 (2); Pb.53/99 (1); see S4 Table) for radiocarbon dating were excavated during the 1951 excavation at the site [15], and had been stored at the Hungarian National Museum. The samples were subsequently returned to the museum following analysis. All samples were limb bone fragments of *Alces alces* which comprise ~70% of the total bone assemblage, including the bone tools [45]. The samples were found together with bone mining tools and a number of stone artefacts in layer 5 immediately overlying the bedrock at mining pit number 2 [15].

The samples were pretreated at the Department of Human Evolution, Max Planck Institute for Evolutionary Anthropology (MPI-EVA), Leipzig, Germany, using published methods [79]. The outer surface of the bone samples were first cleaned by a shot blaster and then subsampled for 500 mg of bone powder. The samples were then decalcified in 0.5M HCl at room temperature for approximately 4 hours until no CO_2_ effervescence was observed. 0.1M NaOH was added for 30 minutes to remove humic material. This was followed by a final 0.5M HCl digestion for 15 minutes. The resulting solid was gelatinized following [80] at pH3 in a heater block at 75°C for 20 hours. The gelatine was then filtered in an Eeze-Filter (Elkay Laboratory Products (UK) Ltd.) to remove small (<80 μm) particles. The gelatine was then ultrafiltered with Sartorius “Vivaspin Turbo” 30 KDa ultrafilters [81]. Prior to use the filter was cleaned to remove carbon containing humectants [82]. The samples were lyophilized for 48 hours.

Stable isotopic analyses were undertaken at MPI-EVA, Leipzig (Lab Code S-EVA), using a Thermo Finnigan Flash EA coupled to a Delta V isotope ratio mass spectrometer. For good quality collagen, the C:N ratio should be between 2.9 and 3.6 and the collagen yield no less than 1% of the sample weight [83, 84]. At Lovas the isotopic values, C:N ratios and yield of collagen were fully within the acceptable range (S4 Table).

After all the quality criteria were evaluated, between 3 mg to 5 mg of the collagen extracted were sent to the Klaus-Tschira-AMS facility of the Curt-Engelhorn Centre in Mannheim, Germany (Lab Code MAMS), where they were graphitized and dated [85].

The resulting radiocarbon dates are listed in Table 1. All dates were corrected for residual preparation background estimated from pretreated ^14^C free bone samples, provided by the MAMS and pretreated in the same way as the archaeological samples.

## Supporting Information

S1 Fig Detailed X-ray diffractogram of the red ochre. (TIF)

S2 Fig Detailed X-ray diffractogram of the red veins in the dolomite. (TIF)

S3 Fig Detailed X-ray diffractogram of the red ochre <10 μm near a formerly found bone tool (by sedimentation). (TIF)

S4 Fig SEM image of red ochre (<10 μm) next to the bone tool. (TIF)

S5 Fig Sub-micron hematite particles on rhombohedral dolomite crystal surface. (TIF)

S1 Table Semi-quantitative data of the mineral phases and major element composition of red ochre. (DOCX)

S2 Table Semi-quantitative data of the mineral phases and major element composition of red ochre (<10 μm) next to the bone tool. (DOCX)

S3 Table Chemical component of red ochre (by energy-dispersive spectrometer). (DOCX)

S4 Table Isotopic values, C:N ratios, amount of collagen extracted (%Coll) refer to the >30 kDa fraction. The results of AMS radiocarbon dating of 5 samples from Lovas. δ13C values are reported relative to the vPDB standard and δ15N values are reported relative to the AIR standard. The human modified bones are indicate by an asterisk (*) on the MPI Lab Code.
(DOCX)
