# Supplementary figures and images for: Core-Shell Processing of Natural Pigment: Upper Palaeolithic Red Ochre from Lovas, Hungary
Source: PLoS One. 2015 Jul 6;10(7):e0131762. doi: 10.1371/journal.pone.0131762 (PMC4509578; doi:10.1371/journal.pone.0131762)

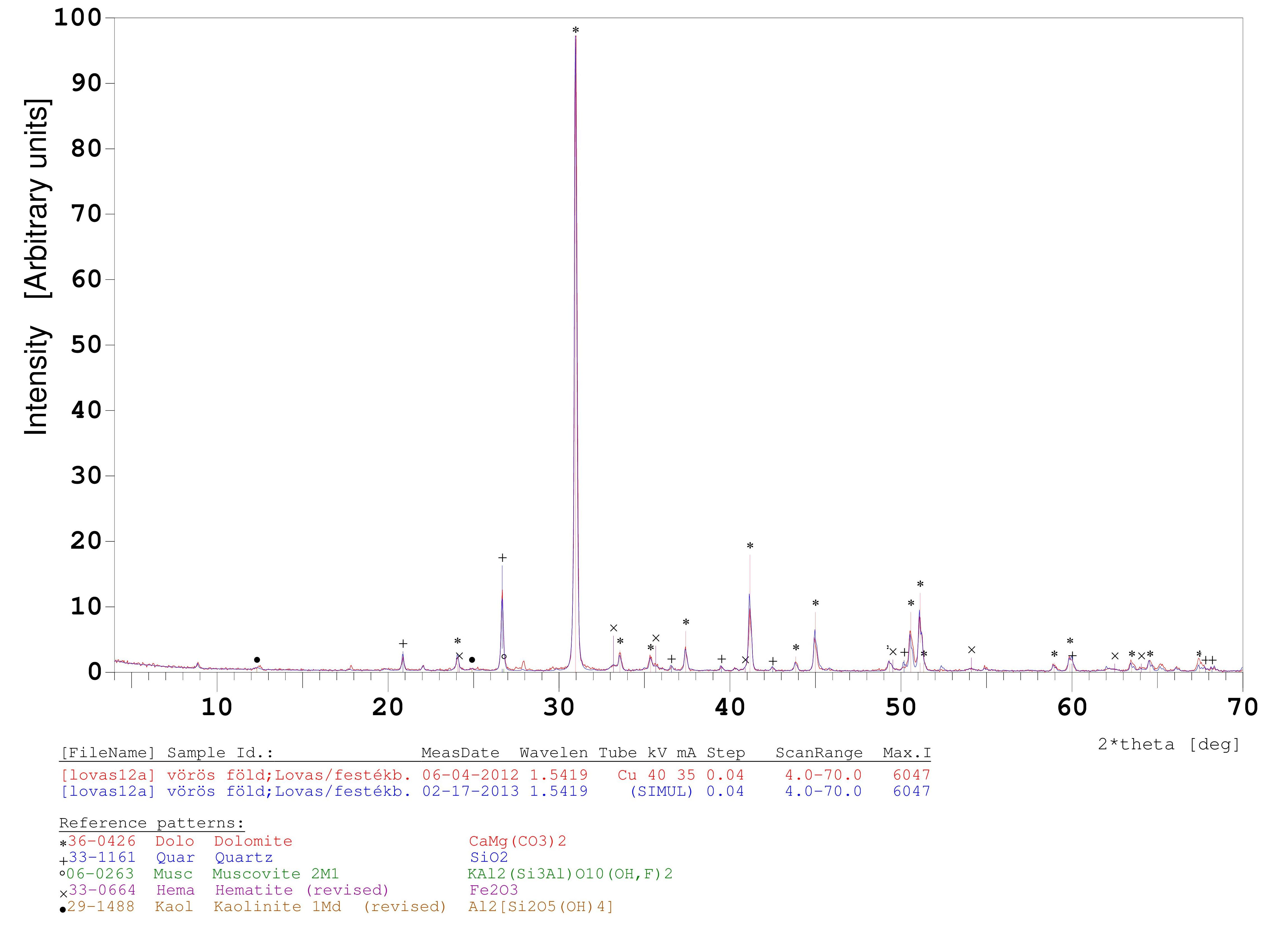

Supplement: S1 Fig — (TIF) [file pone.0131762.s001.tif]

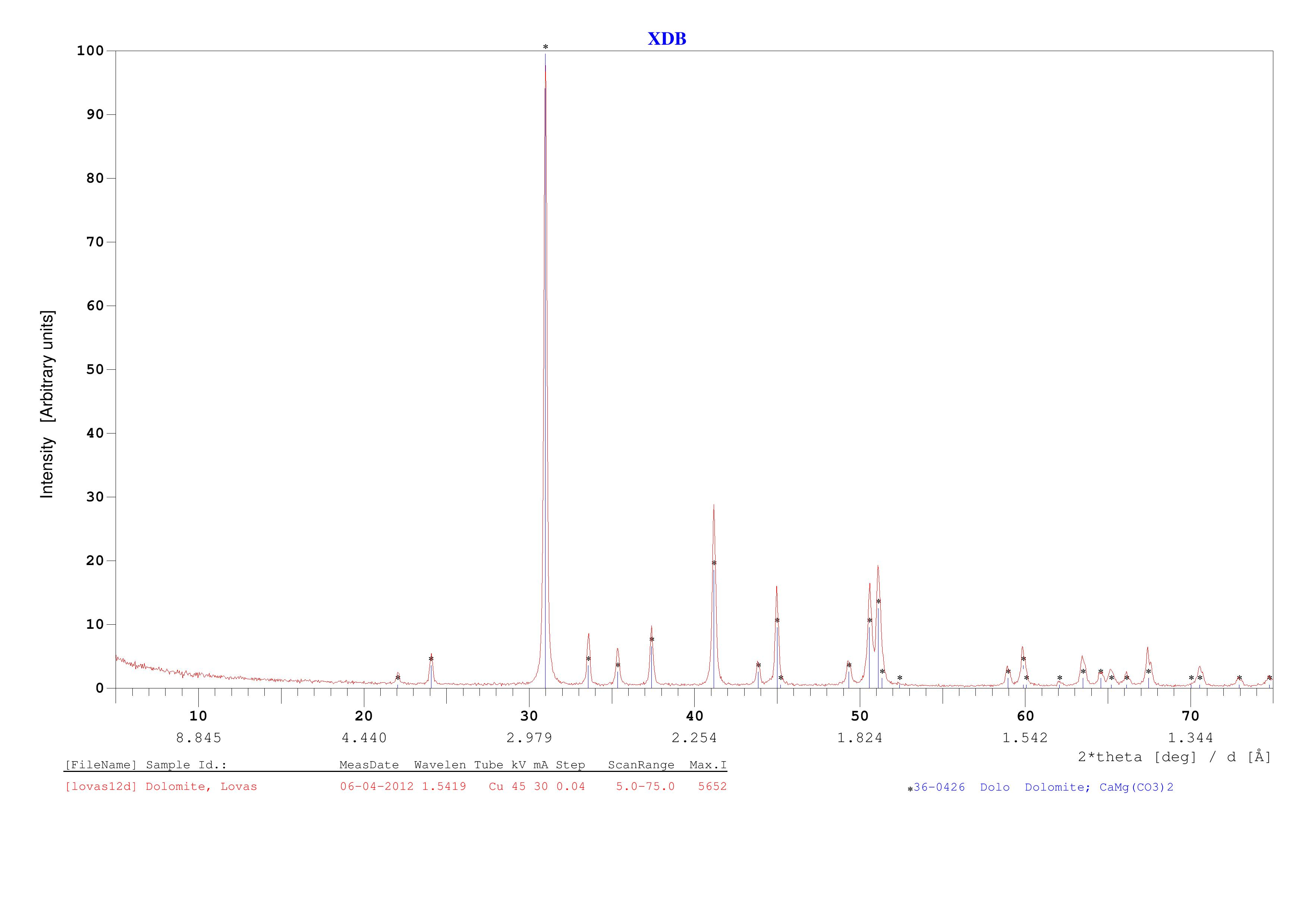

Supplement: S2 Fig — (TIF) [file pone.0131762.s002.tif]

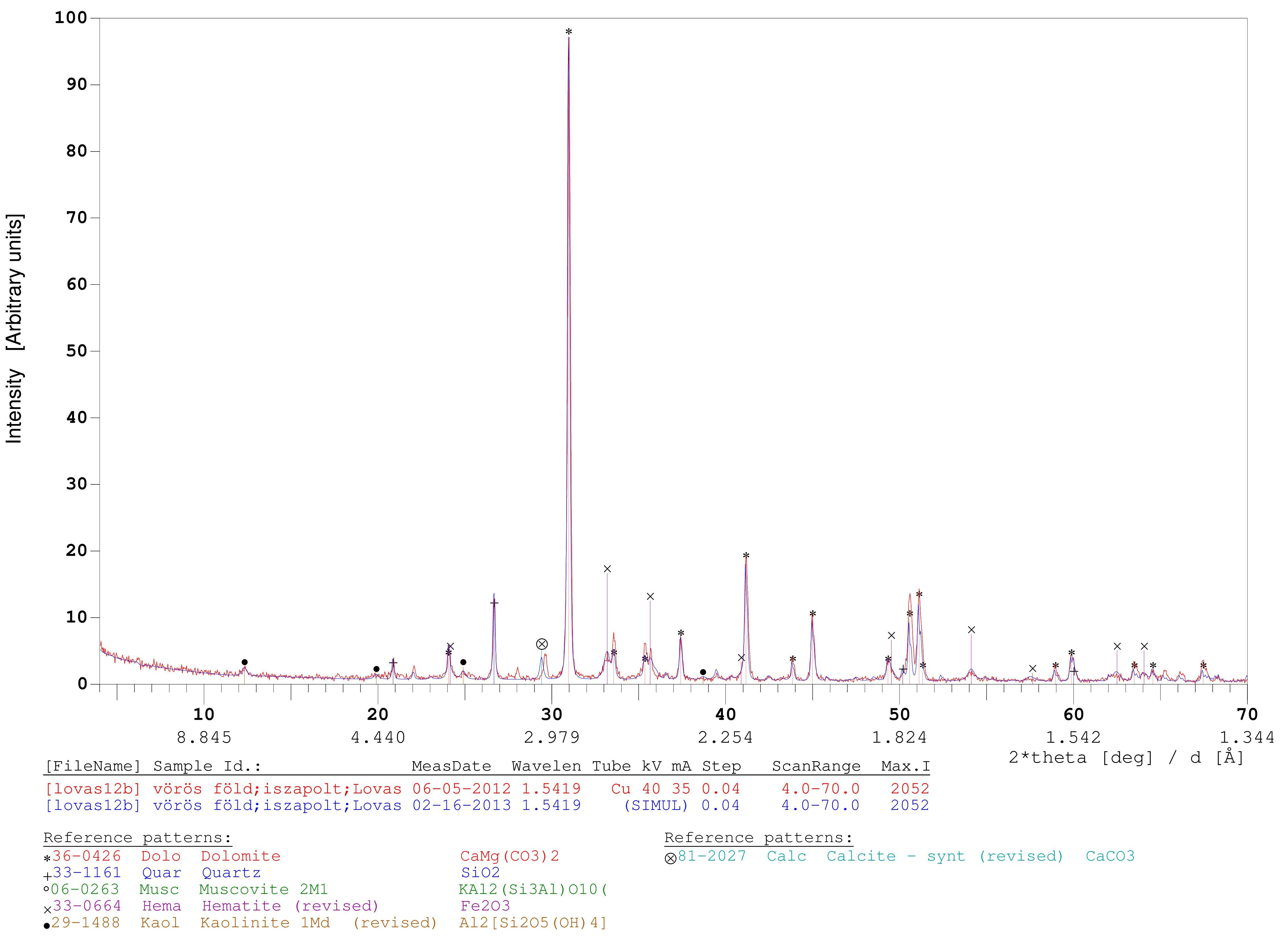

Supplement: S3 Fig — (TIF) [file pone.0131762.s003.tif]

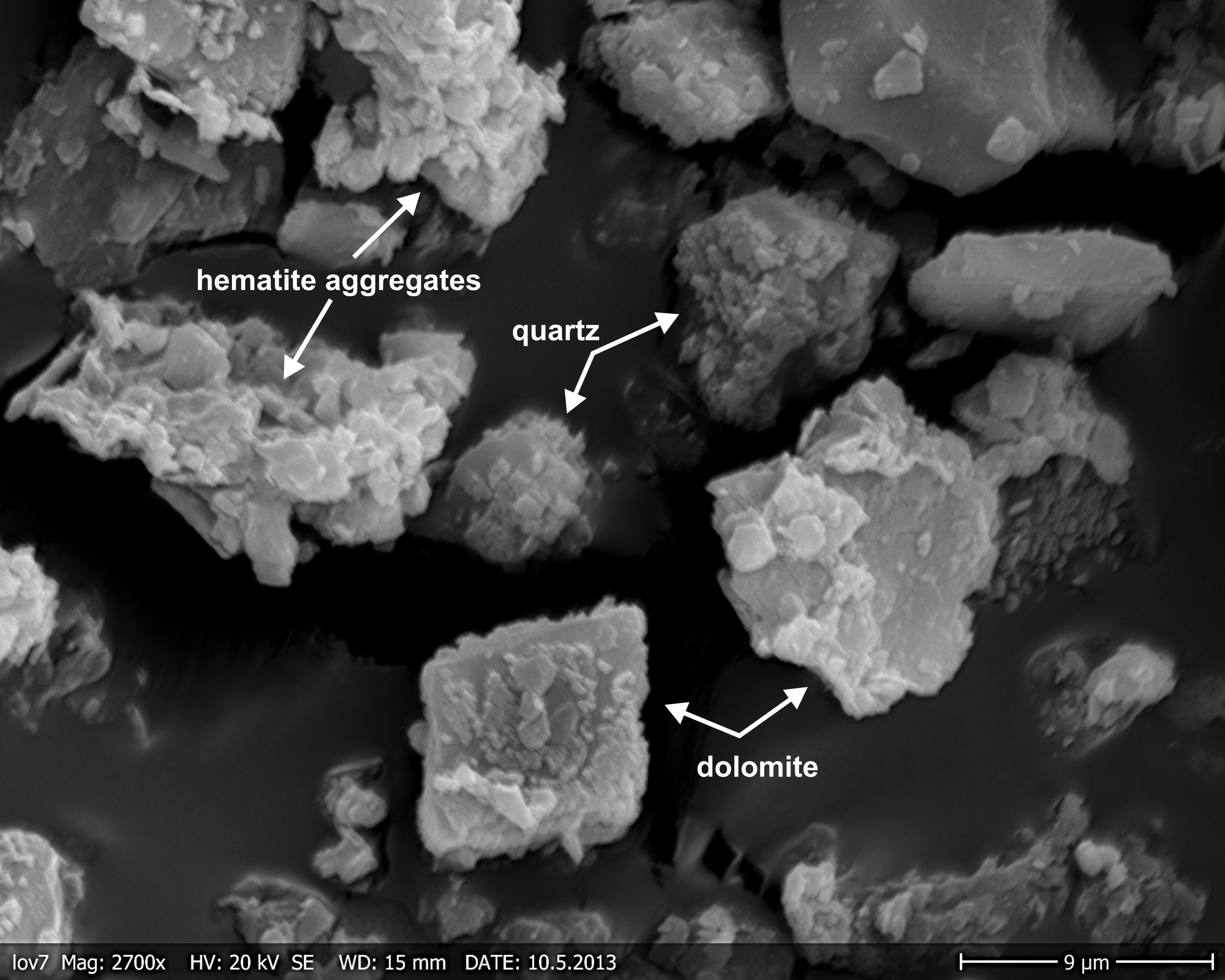

Supplement: S4 Fig — (TIF) [file pone.0131762.s004.tif]
