# Supplementary material for: Core-Shell Processing of Natural Pigment: Upper Palaeolithic Red Ochre from Lovas, Hungary
Source: PLoS One. 2015 Jul 6;10(7):e0131762. doi: 10.1371/journal.pone.0131762 (PMC4509578; doi:10.1371/journal.pone.0131762)
Supplement: S1 Table — (DOCX) [file pone.0131762.s006.docx]

**S1 Table. Semi-quantitative data of the mineral phases and major element composition of red ochre**

|  | **SUM** | **Dolomite** | **Quartz** | **Muscovite** | **Hematite** | **Kaolinite** |
| --- | --- | --- | --- | --- | --- | --- |
| **Phase(%)** | 100.00 | 80.00 | 10.00 | 1.00 | 5.00 | 4.00 |
| **Fe_2_O_3_** | 5.00 |  |  |  | 5.00 |  |
| **CaO** | 24.33 | 24.33 |  |  |  |  |
| **K_2_O** | 0.12 |  |  | 0.12 |  |  |
| **SiO_2_** | 12.31 |  | 10.00 | 0.45 |  | 1.86 |
| **Al_2_O_3_** | 1.96 |  |  | 0.38 |  | 1.58 |
| **MgO** | 17.49 | 17.49 |  |  |  |  |
| **F** | 0.02 |  |  | 0.02 |  |  |
| **CO_2_** | 38.19 | 38.19 |  |  |  |  |
| **H_2_O** | 0.59 |  |  | 0.03 |  | 0.56 |
| **LOI** | 38.78 | 38.18 | 0.00 | 0.04 | 0.00 | 0.56 |
