# Supplementary material for: Core-Shell Processing of Natural Pigment: Upper Palaeolithic Red Ochre from Lovas, Hungary
Source: PLoS One. 2015 Jul 6;10(7):e0131762. doi: 10.1371/journal.pone.0131762 (PMC4509578; doi:10.1371/journal.pone.0131762)
Supplement: S2 Table — (DOCX) [file pone.0131762.s007.docx]

**S2 Table. Semi-quantitative data of the mineral phases and major element composition of red ochre (<10 µm) next to the bone tool**

|  | **SUM** | **Dolomite** | **Quartz** | **Calcite** | **Hematite** | **Kaolinite** |
| --- | --- | --- | --- | --- | --- | --- |
| **Phase(%)** | 99.00 | 70.00 | 6.00 | 3.00 | 12.00 | 8.00 |
| **Fe_2_O_3_** | 12.00 |  |  |  | 12.00 |  |
| **CaO** | 22.97 | 21.29 |  | 1.68 |  |  |
| **SiO_2_** | 9.72 |  | 6.00 |  |  | 3.72 |
| **Al_2_O_3_** | 3.16 |  |  |  |  | 3.16 |
| **MgO** | 15.30 | 15.30 |  |  |  |  |
| **CO_2_** | 34.73 | 33.41 |  | 1.32 |  |  |
| **H_2_O** | 1.12 |  |  |  |  | 1.12 |
| **LOI** | 35.85 | 33.41 | 0.00 | 1.32 | 0.00 | 1.12 |
