# Supplementary material for: Core-Shell Processing of Natural Pigment: Upper Palaeolithic Red Ochre from Lovas, Hungary
Source: PLoS One. 2015 Jul 6;10(7):e0131762. doi: 10.1371/journal.pone.0131762 (PMC4509578; doi:10.1371/journal.pone.0131762)
Supplement: S3 Table — (DOCX) [file pone.0131762.s008.docx]

**S3 Table. Chemical component of red ochre** (by energy-dispersive spectrometer)

| **Component** | **Concentration** | **Conc. Error** | **Units** |
| --- | --- | --- | --- |
| **Mg** | 4.27 | 1.19 | wt.% |
| **Al** | 3.75 | 0.65 | wt.% |
| **Si** | 6.82 | 0.56 | wt.% |
| **S** | 0.40 | 0.07 | wt.% |
| **Cl** | 0.60 | 0.06 | wt.% |
| **K** | 7.30 | 0.30 | wt.% |
| **Ca** | 52.12 | 0.74 | wt.% |
| **Ti** | 0.58 | 0.07 | wt.% |
| **V** | 0.45 | 0.05 | wt.% |
| **Cr** | 0.17 | 0.03 | wt.% |
| **Mn** | 0.71 | 0.05 | wt.% |
| **Fe** | 22.77 | 0.30 | wt.% |
