# Supplementary material for: Core-Shell Processing of Natural Pigment: Upper Palaeolithic Red Ochre from Lovas, Hungary
Source: PLoS One. 2015 Jul 6;10(7):e0131762. doi: 10.1371/journal.pone.0131762 (PMC4509578; doi:10.1371/journal.pone.0131762)
Supplement: S4 Table — The results of AMS radiocarbon dating of 5 samples from Lovas. δ13C values are reported relative to the vPDB standard and δ15N values are reported relative to the AIR standard. The human modified bones are indicate by an asterisk (*) on the MPI Lab Code. (DOCX) [file pone.0131762.s009.docx]

**Radiocarbon Dating**

**S4 Table. Isotopic values, C:N ratios, amount of collagen extracted (%Coll) refer to the >30 kDa fraction.** The results of AMS radiocarbon dating of 5 samples from Lovas. δ^13^C values are reported relative to the vPDB standard and δ^15^N values are reported relative to the AIR standard. The human modified bones are indicate by an asterisk (*) on the MPI Lab Code.

| **MPI Lab Code** | **Submitter No.** | **Species** | **Element** | **Coll. %** | **δ^13^C** | **δ ^15^N** | **%C** | **%N** | **C:N** | **AMS Lab Code** | **^14^C Age** | **Err 1σ** |
| --- | --- | --- | --- | --- | --- | --- | --- | --- | --- | --- | --- | --- |
| S-EVA 29908* | Pb.53/96.3 (100) | *Alces alces* | postcranial | 7.7 | -21.0 | 5.8 | 45.4 | 15.9 | 3.3 | MAMS-21718 | 11,941 | 44 |
| S-EVA 29909* | Pb.53/98.2 | *Alces alces* | postcranial | 3.6 | -21.4 | 4.6 | 37.5 | 12.8 | 3.4 | MAMS-21719 | 11,918 | 41 |
| S-EVA 29910* | Pb.53/97.3 (157) | *Alces alces* | postcranial | 5.5 | -20.9 | 3.1 | 42.9 | 14.9 | 3.4 | MAMS-21720 | 11,825 | 41 |
| S-EVA 29912* | Pb.53/99 (2) | *Alces alces* | postcranial | 1.7 | -21.8 | 3.2 | 43.2 | 14.5 | 3.5 | MAMS-21722 | 11,728 | 46 |
| S-EVA 29911* | Pb.53/99 (1) | *Alces alces* | postcranial | 2.4 | -20.9 | 3.8 | 43.6 | 15.3 | 3.3 | MAMS-21721 | 11,469 | 40 |
| - | L.1758 | *Alces alces* | - | - | - | - | - | - | - | ETH-15119 | 11,740 | 100 |
